# Abstract of the Proceedings of the Racine Medical Association

**Published:** 1863-04

**Authors:** John D. Meachem

**Affiliations:** Secretary; Racine, Wis.


ART. II.—*Abstract of the Proceedings of the Racine Medical Association.*

The Association met at the office of Dr. Thompson on Tuesday evening, March 9th.

Present—Dr. Page in the Chair; Drs. Smith, Nettleton, Hoy, Wadsworth, Thompson and Meachem.

*Dr. Wadsworth* read a very able paper on venereal abuses, masturbation, &c. He said that the first great lesson that should be learned by all, was knowledge of the organic laws. He who conforms to them, and moves in harmony with their purposes, in all his ways, secures health and happiness as his sure reward; but woe to the man who through ignorance or defiance, contends with, or disobeys these laws. Disease and suffering will meet him on every side, turning all his pleasures into pain, and his strength into weakness, wretchedness, and often premature death.

After the reading of the essay, the Chair proposed Pneumonia as a subject for the evening discussion.

*Dr. Wadsworth* said he should like to hear from Dr. Meachem in relation to the treatment of pneumonia. as he had recently seen a case in consultation with him, at the military hospital, that he had treated almost wholly by the use of stimulants and liberal diet.

*Dr. Meachem* stated that his plan of treating pneumonia was very different from that practiced by him a few years ago. In the early part of his professional life, he not unfrequently bled his patients three or four times during the attack of the disease. He now seldom bled, except locally. He would not denounce general bleeding altogether, for he could now imagine cases when he would resort to it, though he believed he had not, in pneumonia, for the last three years. If called to a case early, and cathartics had not previously been administered, (which is not often,) he gave calomel and rhuebarb, or some of the salines, sufficient to produce two or three pretty free dejections; after which he prescribed the tincture of veratrum viride, in doses large enough to bring the system fully under its influence. He could control the pulse by this remedy, far better than he could by general bleeding, and his patients convalesced more speedily. He had never seen any unpleasant consequences follow its use, for after its full effect had once been produced, it required but very little to keep up the impression as long as desirable. He had great confidence in blistering, if not resorted to, too early in the disease. Many practitioners make a great mistake in this respect. They will apply blisters in the second or third day, which almost invariably aggravate the case. He had a recent conversation with the Chairman, Dr. Page, on this subject, who informed him that he had seen simple cases of pneumnoia, converted into very severe ones, by the untimely application of even mustard cataplasms.

The old plan of administering calomel and opium, day after day, until the specific effect of the mercury was visible in the mouth, he denounced altogether. Occasional anodynes, are many times very beneficial, and the same may be said of mercury, but he did not believe in its being administered with the view to the constitutional effect. He had heard physicians say that they had never lost patients of pneumonia, in whom salivation was produced. How many they lost in *trying* to produce it, and in whom they did not succeed, was a matter, he feared, upon which their memories did not care to dwell. Most patients with pneumonia, after the first three or four days, will bear stimulants well, and in many instances with marked benefit.

*Dr. Thompson* had too often seen the good effects of bleeding, to feel safe in neglecting it in severe cases of pneumonia. He thought twenty years ago that it was practiced too much, and he now thinks it is practiced too little. He did not believe in extremes either way. He was quite sure that patients were more perfectly cured, and had less chronic troubles remaining, who had been liberally bled during the early stages of the disease.

*Dr. Wadsworth* remarked, that he thought it very important to make a correct diagnosis, and especially of the different stages of the disease.—This only would be a safe guide to the treatment. In the first stage, that of engorgement, was the time for depletion. The lancet, if used at all, must then be employed. Latterly he had not resorted much to it, but had used the veratrum viride instead, and thought it a valuable remedy in the disease. When consolidation or hepatization is present, he avoided direct depletion, and instituted a supporting treatment. Calomel and opium he considered eminently useful. He was aware that it had been said by the *moderns* that the opium was all that was beneficial in the prescription, and of course calomel went by the board. He could not subscribe, but even protested against this doctrine, for in a practice of more than thirty years, he had not seen a single death by pneumonia, when the specific effect of the mercury was obtained. His convictions were therefore strongly in its favor and he could not conscientiously give up the remedy. He had often, in the latter stages of the disease, had recourse to stimulants. The idea of inflammation would not retard him from their use, and especially in the case of children. He was satisfied that he had saved the lives of many children by this means, that otherwise would have been lost. Finally, he urged upon the members of the Association the study of auscultation, as giving precise knowledge that would enable us to adapt our treatment to the different stages, with more accuracy and success.

*Dr. Page* formerly extracted blood, either locally or generally, in the first stage of pneumonia. Of late years he had trusted to veratrum viride, except in persons of full habit, or when the congestion threatened sudden lesion of the lungs, in which case he still practiced venesection. He had been in the habit of prescribing mercury cautiously—usually in combination with opium. After the first stage, when the heat of skin had lessened and the pulse softened, he frequently applied blisters. Sinapisms (usually applied before the physician is called) often do much harm. Quinia is usually indicated in malarial districts; and in adynamic cases stimulants are required—sometimes from the beginning. He had bled to relieve local engorgement, and the next moment prescribed stimulants. The function of the kidneys should be looked after; and diuretics are frequently demanded. He who never bleeds, (although he may do less mischief,) is as much, in error, as he who always bleeds. He considers Bennett's plan of treating pneumonia, as altogether too inefficient, except in mild cases.

*Dr. Nettleton* remarked that the name of this, as well as of other diseases, was too often treated, and not the condition of the patient. The state of the system is of more importance than the name of the malady. It depends upon the judgment of the medical attendant, to decide the existing present state of the system, and stage of the disease, and to direct his remedial means adapted to the condition of the patient at the time.—The most appropriate remedies, in his estimation, in the treatment of pneumonia are—1st, Venesection; 2d, Tartar emetic, not to nauseate; 3d, Opium in large doses; and 4th, Ipecac, Polygala Senega, &c. In all cases warmth and moisture to the chest.

*Dr. Hoy* had used the veratrum in combination with gelseminum, in a recent case of pneumonia, with excellent effect. The patient was a strong robust man, and the attack very severe, but it yielded speedily to the combination of these two remedies.

*Dr. Page* reported a case of syphilitic orchitis, of long standing, and bad management in the hands of quacks. The testicle was removed by Dr. Meachem. It had degenerated into a fibrous mass, weighing ten ounces. The spermatic cord being seriously involved, was ligated high up, and the case progressed favorably with no untoward symptoms.

JOHN D. MEACHEM, M. D., Secretary.

*Racine, Wis.*, March 20, 1863.

---

ART. III.—*Abstract of the Proceedings of the Buffalo Medical Association.*

